# Viruses Challenge Selectivity Barrier of Nuclear Pores

**DOI:** 10.3390/v5102410

**Published:** 2013-09-30

**Authors:** Aksana A. Labokha, Ariberto Fassati

**Affiliations:** The Wohl Virion Centre and MRC Centre for Medical & Molecular Virology, Division of Infection and Immunity, University College London, Cruciform Building, 90 Gower Street, London WC1E6BT, UK

**Keywords:** NPC, importin, exportin, nucleoporin, FG hydrogel, viral capsid

## Abstract

Exchange between the nucleus and the cytoplasm occurs through nuclear pore complexes (NPCs) embedded in the double membrane of the nuclear envelope. NPC permeability barrier restricts the entry of inert molecules larger than 5 nm in diameter but allows facilitated entry of selected cargos, whose size can reach up to 39 nm. The translocation of large molecules is facilitated by nuclear transport receptors (NTRs) that have affinity to proteins of NPC permeability barrier. Viruses that enter the nucleus replicate evolved strategies to overcome this barrier. In this review, we will discuss the functional principles of NPC barrier and nuclear transport machinery, as well as the various strategies viruses use to cross the selective barrier of NPCs.

## 1. Introduction

The double membrane of the nuclear envelope surrounds the nucleus and separates the cellular genome from the cytosol during interphase. This subdivision leads to the physical separation of transcription and translation, which require a highly coordinated exchange between the cellular compartments. Viruses that replicate in the nucleus also have to pass the nuclear envelope barrier during the infection. Almost all exchange between nucleus and cytoplasm occurs through the nuclear pore complexes (NPCs) that are embedded in the double membrane of the nuclear envelope [1,2,3,4,5].

**Figure 1:**
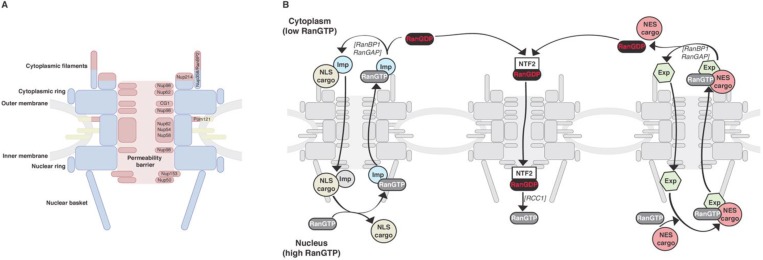
Schematic represenation of metazoan NPC composition and nucleocytoplasmic transport.

NPCs are very large macromolecular assemblies with an approximate mass of 125 MDa in higher eukaryotes [6,7]. This exceeds 25 times the mass of a eukaryotic ribosome [8], which also points to the complexity of its biogenesis. The vertebrate NPC has an eight-fold rotational symmetry and contains multiple structural domains [9,10]. These include cytoplasmic filaments, nuclear and cytoplasmic rings, a scaffold of eight large spikes, a nuclear “basket” and, located in the central channel, the permeability barrier controlling the selectivity and the rate of nucleocytoplasmic exchange (Figure 1A). 

## 2. Nucleocytoplasmic Transport

The NPC functions as a highly selective gate and allows passage of molecules in two modes: passive diffusion and facilitated translocation (reviewed in [4,5]). Passive diffusion across the barrier is typically efficient only for molecules with a mass not exceeding 20–40 kDa [11]. In contrast, passage of larger objects depends on nuclear transport receptors (NTRs; also called Karyopherins) that have the privilege of facilitated NPC passage [4,12]. A single NPC accommodates the mass flow of nearly 100 MDa/s and approximately 10^3^ facilitated translocation events per second [13]. 

The majority of facilitated translocations are mediated by NTRs of the importin-β superfamily (reviewed in [4,14]). These NTRs shuttle between the cytoplasm and the nucleus, bind cargo molecules on one side of the nuclear envelope and deliver them to the other side. According to the directionality of the transport process, NTRs are classified into importins (Imp) or exportins (Exp). Importins are able to recognise classical and non-classical nuclear localisation signals (NLSs) on the cargo molecules and facilitate their translocation from the cytoplasm into the nucleus (Figure 1B). The classical type of NLSs is represented by a mono- or bipartite stretch of basic amino acids (particularly lysine) [15]. Importin-β binds a broad range of cargos bearing classical NLSs via its adapter importin-α (Impα) [16,17,18,19,20,21]. Impα contains the Impβ binding (IBB) domain that mediates formation of a complex between Impβ and Impα [17,18,22]. Impβ can also recognise non-classical NLSs and bind the cargo directly. Exportins bind to the cargos bearing nuclear export signals (NES) in the nucleus and translocate them into the cytoplasm (Figure 1B). NESs contain 4–5 hydrophobic residues characteristically spaced by charged, polar or small amino acids [23,24,25,26]. 

Nucleocytoplasmic transport through the NPCs occurs in a step-wise manner: (i) binding of a cargo molecule to its cognate NTR; (ii) docking of NTR·cargo complex to the NPC; (iii) translocation through the nuclear pore; and (iv) cargo release on the opposite side of the nuclear envelope. The directionality of Imp/Exp-mediated transport processes is determined by the RanGTP gradient across the nuclear envelope [27,28]. Ran’s guanine nucleotide exchange factor RCC1 localises in the nucleus [29] resulting in a high nuclear RanGTP concentration [30]. RanGTP enforces the disassembly of Imp·cargo complexes and promotes the assembly of Exp·cargo·RanGTP complexes inside the nucleus [27,31,32]. Notably, Ran binding sites in Impβ are essential for the termination of import processes [27,33]. Experiments performed with Impβ mutants that lack Ran binding sites showed accumulation of Impβ·cargo complexes on the nucleoplasmic side of the NPCs without further cargo release into the nucleoplasm. After the cargo release is completed, importins return to the cytoplasm in the RanGTP-bound form.

In the cytoplasm, RanGTPase-activating protein RanGAP triggers hydrolysis of Ran-bound GTP [34,35]. However, when sequestered in kinetically stable complexes, GTP-bound Ran resists GTPase activation by RanGAP. Here, the Ran-binding proteins RanBP1 and RanBP2/Nup358 promote the initial dissociation of Ran from the transport factors such that RanGAP can mediate the GTP hydrolysis and trigger complexes disassembly on the cytoplasmic side of NPCs [36,37,38,39]. A major fraction of RanGAP is bound to RanBP2/Nup358 (cytoplasmic fibrils) due to its modification with ubiquitin-related modifier SUMO1 [40,41]. The remaining fraction of RanGAP is soluble in the cytoplasm. A dedicated nuclear import receptor—NTF2—imports RanGDP back into the nucleus [42].

## 3. NPCs

NPCs are composed of approximately 500 individual polypeptides representing multiple copies of about 30 different nuclear pore proteins (nucleoporins, Nups) [43]. According to their localisation and function in NPC biogenesis, vertebrate nucleoporins can be classified into three categories (Figure 1A). The first group includes nucleoporins that contain transmembrane domains and anchor the NPCs into the nuclear envelope. The second group contains approximately 19 nucleoporins that form the rigid NPC scaffold. Finally, the third group is represented by nucleoporins containing globular NPC anchoring domains and non-globular phenylalanine-glycine (FG) rich domains on their N- or/and C-termini. These FG-rich nucleoporin regions face into the central channel of NPCs [44] and are thought to form the permeability barrier that controls nucleocytoplasmic translocations [45,46,47].

The natively unfolded nucleoporin domains are enriched with Phe-Gly (FG), Phe-x-Phe-Gly (FxFG, “x” is any amino acid) or Gly-Leu-Phe-Gly (GLFG) clusters separated from each other by hydrophilic spacer regions of different length. Approximately 1/3 of all nuclear pore proteins are FG nucleoporins [48]. FG nucleoporins directly interact with NTRs via FxFG, GLFG clusters and therefore play an important role in the transport processes across the nuclear envelope [49,50,51,52,53].

A remarkable feature of vertebrate FG nucleoporins is that they are post-translationally modified by the attachment of monomeric residues of O-linked *N*-acetylglycosamine (O-GlcNAc) to Ser or Thr amino acids [54,55,56,57,58]. Recently, it was shown that these modifications are necessary to fine-tune the permeability of NPC barrier [59]. This so far unknown feature might initiate a new direction in research on how viruses explore host enzymes to enchance the glycosylation of FG Nups and thus increase the permeability of NPC barrier, making the translocation of large viral particles easier.

## 4. NPC Barrier

The permeability barrier of the pore plays a dual role: it restricts the diffusion of inert molecules, whose mass exceed 20–40 kDa, but allows the passage of large NTR·cargo complexes, whose mass can reach several megadaltons. The facilitated translocation of NTR·cargo complexes through the NPCs barrier is linked to the interactions between NTRs and FG nucleoporins. Several models have been proposed to explain the molecular mechanism of NPC permeability barrier function. 

The “Brownian/virtual gate” model [60,61] proposes that FG repeat domains are unstructured and mobile on both entry sites of the NPCs creating an entropic barrier. Brownian motion of FG domains deflects large inert molecules from the channel, while the small molecules can slip past FG domains. NTRs can overcome this barrier due to the affinity to FG repeat domains. The diffusion degree of FG domains reduces upon NTRs binding thereby facilitating the passage through the pore. Even though a polymer brush can repel macromolecules through volume exclusion effects, an effective repulsion would require an extremely high grafting density (much higher than within an FG meshwork) and it would fade out with increasing distance from the anchoring points. In this context, it is interesting to note that the deletion of approximately half of the FG mass from the NPCs does not lead to the collapse of the permeability barrier [62]. 

The “reduction of dimensionality” model [63] assumes that channels in the NPC are so narrow that passage of inert macromolecules is essentially suppressed. NTR bound material is then thought to slide along the channel walls (lined with FG repeats) and to reach, by a 1D random walk, the other side faster than inert material by 3D-diffusion. The problem is that channels that are narrow for ribosomes will appear “wide” for GFP-sized objects. This model therefore fails to explain why NPCs are at the same time selective for small-sized objects (diameter ≈ 5 nm) and for ribosome-sized objects (diameter 25 nm).

The “selective phase” model was proposed to explain the molecular mechanism of NPC permeability barrier function. The model assumes that the interactions between the hydrophobic FG-clusters crosslink the FG-repeat domains into a sieve-like FG-hydrogel [13,45]. Accordingly, the mesh size of the sieve allows the free diffusion of small molecules up to 30 kDa, whereas larger molecules are excluded from passive translocation. Nevertheless, experimental data show that NTRs and their cargo complexes, which exceed this size limit, can efficiently traverse the NPCs. Most likely, NTRs facilitate their movement into the barrier by their ability to open up the meshes during the interactions with the hydrophobic FG clusters [46]. Experiments in permeabilised HeLa cells [45] supported the hydrophobic character of inert-FG-repeat interactions. Recent study using reconstituted nuclei from *Xenopus* egg extract and recombinant FG Nups demonstrated a requirement for multivalent cohesion between FG repeats to maintain integrity and selectivity of NPC barrier [64]. This cohesiveness is tuned to promote rapid assembly of the permeability barrier and to generate a stable pore-filling meshwork with a small mesh size [65]. Experiments with Nsp1 nucleoporin showed that, at sufficiently high density, it forms a hydrogel that recapitulates the permeability barrier found in NPCs [66]. In addition, solid-state NMR spectroscopy with yeast Nsp1 FG hydrogel revealed inter-molecular beta-sheet formation involving the Thr, Asn and Gln residues located in the spacer regions between FG clusters [67]. Experiments with permeabilised HeLa cells showed the heterogeneity of mesh sizes with the prevalence mesh radius of ≈ 2.6 nm [11]. The concept of “selective phase” model was supported by the experiments showing that the translocation of cargos through the NPCs barrier slows down with the increase of polar surface. However, when polar residues of cargo molecules are masked by NTRs the translocation through the NPC barrier increases despite the increased mass of the complexes [45]. Thus, the relative influx rate for the same cargo transported by either Impβ or Transportin is 0.28 and 0.18, respectively. In the case of the translocation being facilitated simultaneously by Impβ and Transportin, the relative influx increases up to 3.2 [45]. Thus, the main sorting criterion of NPCs’ barrier is partitioning into a selective phase that is a good “solvent” for NTRs, but not for polar inert macromolecules.

## 5. Viral Nuclear Import: A Brief Overview

Viruses that replicate in the nucleus have evolved strategies to go across the NPC. The variety of strategies developed by viruses to enter the nucleus is remarkable, and likely reflects the need to complete earlier steps such as entry, trafficking and uncoating in an orderly fashion before engagement with NPCs. For example, viruses with large genomes are unlikely to shed their capsid shell early post-infection in the cytoplasm, because the intracellular viral complex would become too bulky and too loose for cytoskeletal transport. Indeed herpesviruses and adenoviruses appear to dock their intact capsid shell at the NPC where partial uncoating occurs, and the viral DNA genome is made visible to the nuclear import machinery [68]. Herpesviruses also appear to exploit a new membrane-based translocation mechanism used by some inner nuclear envelope proteins. This is a vesicular-type of transport occurring at or in proximity of the NPC but without actual translocation across the central channel, which herpesviruses can use to egress from the nucleus [69,70]. Conversely, Influenza viruses have evolved different strategies to engage with NPCs, rapidly recruiting NTRs on their ribonucleoprotein complexes following endosome acidification, fusion and early uncoating in the cytoplasm [68]. Other viruses, such as adeno-associated viruses (AAV) are very small and their intact capsid shell is able to go across NPC [71]. Nuclear import of viral genomes may be important to evade pattern recognition receptors of the innate immune system, found associated with cellular membranes or in the cytoplasm [72]. This likely impacts on the kinetics of nuclear import and indeed there is evidence that some viruses have exploited cellular pathways for the rapid nuclear import of endogenous and exogenous DNA to reduce activation of the innate immune system [73,74].

Another relevant constraint determining the mechanism of viral nuclear import is the size of the viral capsid shell, which often exceeds the functional diameter of the NPC. Studies employing gold nanoparticles determined that 25 nm was the maximal functional diameter of the NPC [75]. However subsequent studies showed that in rare circumstances objects as large as 35–39 nm across can be imported through NPCs [76,77]. Strikingly, tracking large (≈30 nm) quantum dots particles coated with NTRs with super-resolution microscopy showed that they “explore” the central NPC channel in a sub-diffusive fashion and indicated that the overall explorable area of the channel is 55 nm wide and 68 nm long albeit the movement of each quantum dot is limited by molecular crowding inside the channel [77]. In agreement with this data, intact baculovirus core particles of some 35–40 nm in diameter have been detected by electron microscopy to go across NPCs (reviewed in [78]). 

In light of these observations, an important question is how large viral capsids can rearrange FG Nups inside the central NPC channel to negotiate the barrier and at the same time maintain the barrier selectively permeable. One possible model predicts that FG Nups exist in two bi-stable conformations: either clustered towards the centre or towards the NPC wall, depending on the strength of the intermolecular interactions (high strength = centre, lower strength = wall) [79]. Large viral capsids, by virtue of their direct or indirect (mediated by NTRs) interactions with FG Nups, would induce a substantial shift in the strength of intermolecular interactions, causing a local and partial collapse of FG Nups towards the wall and hence allowing very large cargos to pass [79]. Alternatively, by recruiting NTRs or by direct binding to FG-nups, viral complexes may cause local disengagement of inter-repeat contacts, maintaining the permeability barrier at all times during translocation [46]. One prediction for both models is that viral capsids must be densely coated with NTRs, or able to directly engage with multiple FG Nups, in order to change Nups intermolecular interactions. This is consistent with the modular structure of viral capsids, composed of regular repetitive domains, which presumably facilitate their homogeneous coating by NTRs. It is reasonable to predict that interactions between viral capsids and Nups, whether direct or indirect, must be transient and relatively low affinity to allow their detachment from NPCs and delivery to the nucleus.

The capsid of some viruses are able to go across NPC more or less intact, suggesting that uncoating can happen in both cytoplasm and nucleus. Indeed there is evidence that adeno-associated virus (AAV), hepatitis B virus (HBV) and Baculoviruses uncoat after nuclear entry or at the nuclear side of NPCs [71,80,81], and some retroviruses also appear to uncoat in the nucleus, although their capsid enters the nucleus only after breakdown of the nuclear envelope during mitosis [82,83]. HIV-1 has been reported to complete in the nucleus the uncoating process that starts in the cytoplasm [84]. Little is know about factors inducing nuclear uncoating; the NTR Transportin 3 has been proposed to induce completion of HIV-1 uncoating by binding to residual capsid proteins associated with the pre-integration complex inside the nucleus [84]; Impβ and Nup153 appear to stimulate uncoating of HBV at the NPC nuclear basket [81]. In other cases, such as adenovirus type 2, disassembly of the viral capsid seems to occur at the NPC in two steps: first, the core binds to Nup214, then kinesin-1 binds to Nup358 and disassembles the core *in situ* to expose the nucleic acids [85,86]. The viral core protein VII, Transportin 1 and Hsp90 have also been implicated in the nuclear translocation of adenoviral DNA. Herpes simplex virus 1 (HSV-1) capsid is docked to the pore by binding to Nup214 and Nup358, then Impβ and possibly other cellular factors appear to stimulate the disassembly of a single vertex of the capsid, exposing the viral nucleic acids [87,88,89,90].

Viruses that uncoat their capsid shell in the cytoplasm or at the cytoplasmic side of the NPC must have evolved ways to translocate their nucleic acids across the NPC barrier. This is particularly impressive for the large >150 Kb DNA genomes of herpesviruses. Nucleic acids are negatively charged and hydrophylic, which makes them very unsuited to cross the hydrophobic barrier in the NPC central channel. Thus it is not clear how large viral genomes can cross the NPC barrier but it is possible that at least some mechanisms are conserved with export of mRNA complexes (reviewed in [91]). Messenger RNPs have been observed crossing the NPC as large condensed rod-shaped particles in *Chiromonus* salivary glands [92], presumably to limit contacts between hydrophobic surfaces of FG Nups and hydrophylic nucleic acids. In agreement with these early observations, HSV-1 DNA was also detected by atomic force microscopy to engage the NPC as a large rod-like condensed particle [93]. Moreover, mRNA export depends on specific helicases to unwind the mRNA ribonucleocomplex once it has reached the cytoplasmic surface [94]. Currently there is limited information on the structural conformation that viral DNA genomes take during NPC translocation and if motors such as helicases are required to pull them into the nucleus. 

Viruses have evolved to exploit a wide range of host factors for nuclear import, although some pathways appear at least partially conserved and in some cases redundant. For example HIV-1 utilizes Nup358, Nup153, Nup98, in addition to NTRs such as Imp7, Impβ and Impα [95,96,97,98,99,100,101]. Adenovirus type 2 utilizes Nup214, Imp7, Impβ, Impα and Transportin 1 [85,102]. The viral core protein VII, Transportin 1 and Hsp90 have also been implicated in the nuclear translocation of adenoviral DNA [103,104]. HSV-1 exploits Nup358 and Impβ [87,89], influenza viruses have evolved to exploit different Impα isoforms in a cell-specific way [105], and HBV depends on Nup153 and Impβ [81]. In some cases such host factors bind directly to NLSs or specific domains present on viral proteins, in other cases binding is mediated by additional host factors; for example adenovirus type 2 recruits histone H1 to bind Imp7 and Impβ and HIV-1 was shown to exploit tRNAs incorporated into its capsid to promote its translocation across the NPC [106]. It makes sense that viruses have evolved to exploit existing cellular pathways to maximise efficiency of nuclear import. Viruses have also evolved to subvert nuclear import pathways to their advantage. Adenoviruses increase the permeability of NPCs by displacing Nup214, Nup358 and Nup62 to facilitate nuclear import of its ≈30 Kb genome [86]; HIV-1 infection was shown to induce re-distribution of Nup62 to facilitate nuclear export of its genomic mRNA (reviewed [111]). Several viruses, including VSV, Polioviruses, Influenza A, Adenovirus, Herpesviruses disrupt nuclear transport processes to their advantage (reviewed in [91]).

Viruses exploit or alter nuclear transport processes using a remarkable variety of mechanisms, and therefore they have been intensively studied to clarify the biology of nuclear transport [31,106,107,108,109,110]. In this Special Issue of *Viruses*, many aspects of viral nucleocytoplasmic transport will be discussed in greater depth. We hope this Special Issue will provide a useful and enjoyable source of information and stimulate further research in the field.
